# Women’s experiences of factors that facilitate or inhibit gestational diabetes self-management

**DOI:** 10.1186/1471-2393-12-99

**Published:** 2012-09-18

**Authors:** Mary Carolan, Gurjeet K Gill, Cheryl Steele

**Affiliations:** 1 School of Nursing and Midwifery, St Alban’s Campus, Victoria University, PO Box 14228, Melbourne, 8001, Australia; 2 Australian Community Centre for Diabetes (ACCD), Victoria University, St Alban’s Campus, PO Box 14228, Melbourne, 8001, Australia; 3 Western Health, Diabetes Education Service, Western Hospital, Gordon St. Footscray, Victoria, 3011, Victoria, Australia

**Keywords:** Gestational diabetes, Disadvantaged, Barriers, Self-management

## Abstract

**Background:**

Gestational diabetes rates have increased dramatically in the past two decades and this pattern of increase appears to relate primarily to the obesity epidemic, older maternal age and migration from world areas of high GDM risk. Women from disadvantaged and migrant backgrounds are most at risk of developing and of mismanaging this condition. The aim of the study was to explore the factors that facilitated or inhibited gestational diabetes self-management among women in a socially deprived area.

**Methods:**

Fifteen pregnant women, with a diagnosis of gestational diabetes, were purposively recruited for this study. Qualitative semi structured interviews and 1 focus group were conducted when participants were approximately 28–38 weeks gestation. The study’s theoretical framework was based on interpretative phenomenology and data was analysed using a thematic analysis approach.

**Results:**

Women in this study identified a number of factors that complicated their task of GDM self-management. Barriers included: (1) time pressures; (2) physical constraints; (3) social constraints; (4) limited comprehension of requirements, and (5) insulin as an easier option. Factors facilitating GDM self-management included: thinking about the baby and psychological support from partners and families.

**Conclusion:**

Women from low socio economic and migrant backgrounds often struggle to comprehend GDM self-management requirements. To improve adherence to management plans, these women require educational and supportive services that are culturally appropriate and aimed at a low level of literacy.

## Background

Gestational Diabetes Mellitus (GDM), or glucose intolerance that first presents in pregnancy, affects approximately 12,000 pregnant women in Australia annually [1]. This figure represents approximately 4.5-5.0% of all births, although specific groups are at greater risk of developing this disorder [1]. GDM rates have increased dramatically in the past twenty years [2-4] and this pattern of increase appears to relate primarily to the obesity epidemic [5], increasing maternal age [4,6], and migration from high risk areas, such as South East Asia [7]. In Australia, highest rates of GDM are reported among women born in Polynesia, Asia, South Asia (Indian subcontinent) and the Middle East. These populations are at least three times more likely to develop GDM compared to locally-born women [1,8]. Factors such as low socio-economic status and concomitant levels of obesity compound the risk of developing GDM [4].

GDM impacts on the health of both mothers and infants, and gives rise to higher rates of maternal hypertension and pre-eclampsia [9] increased intervention in birth, such as caesarean section [10] and later development of type 2 diabetes [11]. This risk is substantial and women who have had GDM in pregnancy, are at least 6 times more likely to develop type 2 diabetes during their lifetime [11,12]. Gestational diabetes also exposes the fetus to hyperglycemia, which stimulates an increase in fetal insulin and an increased rate of fetal fat storage [6,13]. These two factors in turn predispose the fetus to future obesity and type 2 diabetes. More immediately, the infants of mothers with GDM are more likely to be stillborn [14] or to suffer a range of perinatal morbidities such as birth injuries [15], macrosomia, hypoglycaemia and respiratory problems [10,16]. These morbidities result in higher rates of neonatal nursery admission [16]. Overall, the evidence suggests that women from disadvantaged and migrant communities are the most at risk of both developing GDM [4,17,18] and of misunderstanding and mismanaging the condition [19,20]. Risks of GDM complications are highest for these groups [4,21].

First-line management of GDM involves a complicated self-care regimen of regular blood glucose level (BGL) testing, and dietary adjustment based on the woman’s BGLs. An increase in exercise is also encouraged in a bid to boost the woman’s metabolism [22]. The overall aim of treatment is to maintain BGLs within recommended ranges [22] and this is achieved primarily by reducing energy intake by replacing calorie dense foods with healthier choices [23]. This approach of dietary and exercise adjustment is suitable for approximately 65–90% of women diagnosed with GDM [24-26]. Women with more severe hyperglycaemia and those who are unable to achieve glycaemic goals with diet and exercise require insulin to control their GDM [27]. High levels of insulin administration are a concern however, as women who require insulin to control their GDM are considered to be at higher risk of developing type 2 diabetes in the future [28-30].

In light of these serious implications, for morbidity among both mothers and infants, it is critical that women with GDM are supported to take on the tasks of self-management. This study builds on earlier research which found that knowledge of GDM, food values and GDM management plans was deficient among women in this region [19,20]. This situation of poorer comprehension seemed to relate principally to lower socio economic status, poorer levels of maternal education and lower health literacy (the ability to read and comprehend health related material, such as food labels). The current study sought specifically to understand the factors that facilitated or inhibited women’s understanding and adherence to GDM dietary self-management principles. It was intended as the initial step in the development of an educational and self-management program, aimed specifically at supporting women, with GDM, from disadvantaged and migrant backgrounds.

## Methods

A qualitative approach was chosen to address the complex issues of GDM self-management. This approach was informed by Interpretative Phenomenological Analysis (IPA), as endorsed by Smith and Osborn [31] and Chan et al. [32]. Interpretative phenomenology aims to explore participants’ lived experience of events in order to understand how they make sense of their personal and social worlds (p. 3, 31). The main emphasis is on the exploration of personal experience as the individual narrates his/her account and appraises events [31,32]. This narrative approach also draws on the philosopher Kierkegaard’s [33] insights into the discourses that underpin the lived experience of the narrator in certain situations. Kierkegaard believed that the individual’s stories offered an opportunity for others to see what the storyteller noticed, and to become aware of the particular emphasis he/she accorded to events, including the items that were a concern for him/her [33]. This approach is considered appropriate in this study as it may help uncover the particular concerns and difficulties participants encountered, when self-managing their GDM. The approach is also consistent with an appreciation of the individual woman, which was an important consideration for this study.

Semi structured interviews and one focus group were conducted using a pre-determined set of questions, as below. These questions were intended to loosely guide the interview. A parallel paper, from this study, has reported on the women’s experiences of GDM [34]. This paper reports on the factors that facilitated or hindered the women’s GDM self-management. The study was approved by the Western Health Ethics Committee (Sunshine Hospital). Written consent was obtained prior to interviews and focus group. Pseudonyms were used throughout to ensure the women’s anonymity.

Questions for interview

1. Can you tell me a little about your experience of Gestational Diabetes?

2. 2.Can you tell me a little about the information you received?

3. What other information would you have liked?

4. What made it difficult for you to manage your gestational diabetes?

5. What made it easy for you to manage your gestational diabetes?

6. What management strategies (ways of dealing with your diabetes) worked for you?

7. 7.What advice would you give to someone who was newly diagnosed with GDM

8. What information do you know now, that would have been helpful at the beginning?

### Sample and recruitment

Participants were recruited purposively from a Pregnancy Diabetes Clinic in the Western Region of Melbourne. This clinic serves a socially disadvantaged area with a large multi-ethnic population. Women in this area present with increased risk factors for developing GDM and for poorer GDM self-management, such as low socio-economic status [4,35,36], obesity and poor diet [37], sedentary lifestyle [37], ethnic minority status [4,8] and lower health literacy [19].

Women who met the following inclusion criteria, were invited to participate: pregnant; diagnosis of GDM; able to speak conversational English; singleton pregnancy with no known serious abnormalities. Access to women was facilitated by the diabetes educator, who co-ordinates the women's care at the clinic. GDM testing of participants was consistent with Australasian Diabetes in Pregnancy Society (ADIPS) GDM diagnostic criteria [38], using the 75-g 1 h glucose challenge test (GCT) followed by the 75-g 3 h oral glucose tolerance test (OGTT), if the GCT is positive. A universal approach to screening was employed, as is usual in Australia [4]. Participants were recruited after they had attended for GDM education and had a minimum of 3 weeks experience of self-managing their condition.

In all, 30 women who met the inclusion criteria, and who indicated an interest in the study, were approached. Of this number, 20 women agreed to participate in the study. However, 5 women were unavailable on the day of interview and the most common reason for declining to participate at this stage, was ‘too busy preparing for baby’. A total of 15 women participated in the study

### Data collection and analysis

Data were collected during audio-recorded focus group and interviews. Participants were offered three choices for participation: (1) focus group in a room adjacent to the clinic, (2) individual interview by phone, (3) face to face interview at a venue of their choice. One focus group discussion was conducted involving 4 women, 10 interviews were conducted by phone and the final interview was conducted at the woman’s home. Although the use of focus groups within phenomenological methods is contested [39,40] the most frequent objection is based on the belief that the ‘essence’ of a phenomenon is best explored by individuals who must describe their experience, without interference [39]. However, others argue that focus groups may permit a detailed examination and interpretation of events by allowing participants to share their experiences and engage in a joint sense making endeavour with the focus group facilitator and other participants [41,42]. The use of focus groups in phenomenology is additionally common in nursing and health studies and is generally justified on the premise that participants who share certain features, can relate to each others comments and share experiences to come to a deeper understanding of the phenomenon [43-45]. We would argue that the use of the focus group, as in this study, added to the data in a similar way, and enhanced rather that inhibited the women’s exploration of their experiences.

Data were analysed using Burnard’s [46] method. The following steps were employed:

 • Interview and focus group data were transcribed to facilitate initial familiarisation with the content

 • Audio-tapes were listened to and transcripts were read several times which allowed for an initial identification of themes. This step involved memo-writing and commentary on content

 • Units of meaning (themes) and values were sought. This involved a transformation of memos and notes into themes

 • Data was classified under broad headings, which involved a clustering of emergent themes and ideas

 • Reliability of analysis was addressed by asking a co-researcher to independently generate a theme list

 • Headings were amended and collapsed as data analysis progressed. This involved a stage of higher abstraction and themes were collapsed and refined as meanings became clearer.

 • Emergent understandings were tested against the data, which involved returning to the transcripts to confirm that the interpretations were true to the data

 • Alternate explanations were sought

## Results

Participants came from the following self-identified ethnic backgrounds: Caucasian (n = 5), Indian (n = 4), Vietnamese (n = 2), Arabic (n = 1), Chinese (n = 1), Cambodian (n = 1), Filipino (n = 1). Additional demographic characteristics are presented in Table 1. These groups are also similar to the largest groups to give birth in Victoria, Australia [47]. Most women (73%) were aged between 30–39 years, with an age-range of 23–40 years. Educational level was lower than the Australian population average, and the majority of women reported High School (Secondary) level (73%), as their highest academic achievement. Four women (27%) reported a non-school qualification, which included 3 women (20%) with a university degree. This figure is lower than the Australian population average of 59% non-school qualification, which includes approximately 25% university degree [48]. Parity varied, although the majority (9 women) were primiparous (60%), one third of participants (5 women) were expecting their second baby (33%) and the final participant was expecting her fifth baby. Eleven women (73%) were dealing with GDM for the first time (see Table 1).

**Table 1:** Demographic characteristics of participants

**Women’s pseudonyms**	**Age**	**Highest education level**	**Occupation**	**Gravida**	**Ethnicity**
1. Lili	34 yrs	University	Financial manager	2	Caucasian
2. Loan	38 yrs	High school	Bank teller	1	Vietnamese
3. Rita	31 yrs	High school	Looking for work	2	Caucasian
4. Tran	30 yrs	High school	Office worker	1	Vietnamese
5. Xioquan	29 yrs	High school	Casino croupier	1	Chinese
6. Prani	30 yrs	High school	Carer	1	Indian
7. Flora	32 yrs	High school	Office worker	1	Filipino
8. Leanne	38 yrs	High school	Receptionist	2	Caucasian
9. Kate	32 yrs	University	Nurse	2	Caucasian
10. Margaret	23 yrs	High school	Stay at home mother	2	Caucasian
11. Suji	24 yrs	High school	Factory worker	1	Cambodian
12. Leni	34 yrs	University	Nurse	1	Indian
13. Gurtha	34 yrs	Technical college	Husband’s business	1	Indian
14. Fatima	40 yrs	High school	Stay at home mother	5	Arabic
15. Pina	34 yrs	High School	Not working	1	Indian

### Themes

**Figure 1:**
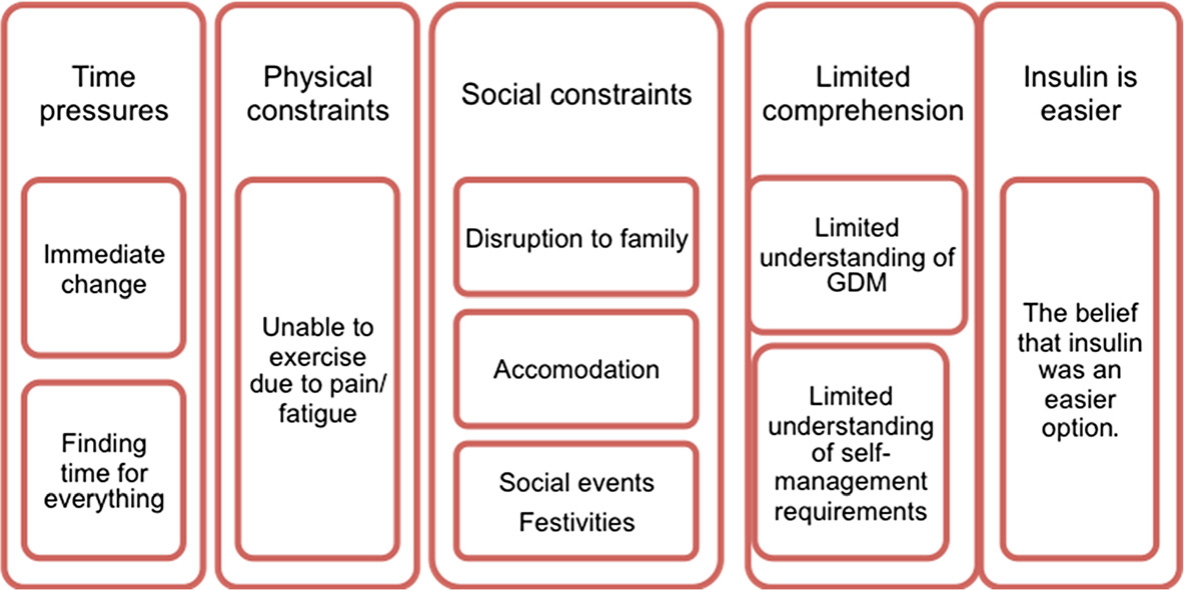
Barriers to GDM self-management.

Women in this study identified a number of factors that assisted or made their task of GDM self-management more difficult. These factors are considered separately under barriers and facilitators of GDM self-management. In this first section, barriers are discussed. The following five themes emerged as barriers: (1) time pressures; (2) physical constraints; (3) social constraints; (4) limited comprehension of requirements, and (5) insulin was an easier option. These themes are illustrated in Figure 1 below:

### Barriers to GDM self-management

#### Theme 1: Time pressures

Participants discussed, at length, the difficulties they encountered when learning to self manage their GDM. Time pressure was identified as possibly the greatest challenge the women faced. This included limited time to understand and make sense of their GDM diagnosis, together with a sense of urgency to effect immediate blood glucose control by adopting the advised dietary regime. Two sub-themes were identified: the urgency of immediate change and finding time for everything.

#### The urgency of immediate change

Most women described a time of shock and confusion as they came to terms with their diagnosis of GDM. This stage was made all the more difficult because of the immediate and quite dramatic dietary change required. Such urgency left participants with what Lili describes as *no time to think it through.* Generally, it took some time for women to make sense of what was required and to understand the seriousness of their condition. Tran describes her experience:

First week, I mean they told me which foods to avoid and so forth, but again that was very limited. I wasn’t eating very much anyway during my pregnancy … quite a few scores [BGL values] were over what they suggested. I hadn’t really looked after what I had been eating…I really wasn’t trying that first week I guess … I didn’t think it would affect the sugar levels so much… She (diabetes educator) thought it was very bad… They suggested insulin after that first week and I didn’t want that at all. I did try to explain, “… I understand why they are high.” I need another chance… Tran

#### Finding time for everything

Finding time for everything was identified as a major challenge for most women. This difficulty related to the busyness of the women’s lives as they juggled work, household chores and family obligations. There were two elements to this sub-theme: dietary self-management and additional requirements. Dietary self-management, in particular, represented an enormous time challenge to women, requiring time to learn about food values in order to create nutritious and appealing meals. For some women, like Leanne, this meant learning to cook, for the first time:

Like where do you find the time… having to go from being able to buy foods (ready cooked) and having to actually think about it, prepare it and cook healthy food. Yeah, lots of processed food (previously)… That’s my biggest change, probably, going from never cooking. Leanne

Most women felt they needed some time to adjust to their new eating regime, and initially, it was difficult to even remember the GDM self-management tasks required. Kate explains:

Because I do shift work, I’ve found I’ve had to make a really strict monitoring sort of system. So I set alarms on my phone every time I have something to eat, so I remember to do the two hours afterwards [BGL], otherwise I’ll just forget. And I set a final alarm to go off before I go to bed so that I can remember to take the night time [insulin]…Kate

Having GDM meant additional requirements such as extra clinic visits to specialists and dieticians. Very often appointments could not be arranged for the woman’s convenience:

Sometimes … I have to actually go back there [clinic] twice a week, I think the dieticians only, you can only book them on a Friday…but the obstetrician, only …on a Monday… so that is hard with work… Flora

Participants also identified finding time to exercise as a particular difficulty, although additional exercise was recommended as part of their diabetes self-management plans. Leanne explains:

… the doctor said to walk for an hour after meals. I mean, I start (work) at seven and finish at three and then I’ve got to pick my daughter up from school. Trying to fit that in, it’s just … I think, well, God, I’ll be dead by the time I get back, you know? … Leanne

#### Theme 2: Physical constraints

The second theme, physical constraints, contains the common explanations offered by women who felt unable to meet with the exercise guidelines of their self-management program. The most common reason offered by women who felt unable to undertake regular walking exercise, as advised by the diabetes educator and midwife, was pain. This pain most often manifested as pelvic (symphysis pubis) pain or backache. Tran describes her difficulty:

Because I have had pelvic pain, I haven’t been able to move a lot. And I have been quite ill… Tran

#### Theme 3: Social constraints

The third theme explores the social constraints identified by participants as creating difficulties for them when self-managing their GDM. There were three sub-themes: disruption to the family, finding the balance, and social events/festivities.

##### Disruption to the family

The first sub-theme encompasses the day-to-day difficulties of having to comply with GDM meal guidelines. Women discuss preparing separate meals for themselves or altering family meals to meet with GDM guidelines. This frequently resulted in a disruption to the family and an additional cost to the family budget.

We had three different dinners every night… I could have eaten the same thing without the carbs… but so boring… I made the choice… and you may not have the budget to buy crazy expensive things… but you have to expect a little bit more… maybe buying organic, interesting vegetables. Do something nice with it, you know, just make mealtimes feel nice… Lili

##### Finding the balance

The second sub-theme illustrates the difficulties participants faced when surrounded by tempting foods at home, or when going to restaurants or visiting family and friends. Although participants were appreciative when family and friends supported them by not having high calorie/high sugar content foods around, they equally did not expect others to entirely change their dietary habits to accommodate the woman’s GDM diet. Xioquan and Suji speak of the difficulties they faced at home:

Because I live with my parents-in-law… Sometimes there is some chocolate or ice cream, sponge cake in the fridge. It is just so hard for me to not touch them…a lot of temptations… Xioquan

I don’t cook the food… it is bad to say I won’t eat (the food that her mother-in-law cooks)… rice… she says it is healthy for the baby… Suji

While Loan found social outings to restaurants, particularly difficult:

Whenever I go to a restaurant with friends that’s the worst case… and especially having the desserts there… But once you go out, like, once a week you want to eat…Loan

Most women describe having small amounts of proffered foods when visiting family and friends. This approach was used so as not to cause offense or difficulty for others, while at the same time trying to adhere to dietary guidelines. Rita explains:

The other week I had a small bowl of pasta… my sugars were very high then. …Because when I’m eating with my Mum… it’s like you can’t expect everybody to change everything… Rita

##### Festivities and social functions

The third sub-theme overviews particular social constraints around special occasions such as religious festivals. Most of these events are accompanied by the provision of high calorie celebratory foods, which are prohibited on GDM self-management plans. Although women are not obliged to eat these foods, food is a large part of the celebration and women felt they missed out when unable to participate in the celebratory meal. Leni explains:

I’m myself Indian and we have lot of Indian sweets and that sort of thing. When I didn’t know that I was diabetic I was eating sweets as well, like Indian proper sweets. And just the religious festivals and eating certain foods, but now I’ve stopped that as well. Actually it’s not compulsory. It’s up to you if you want to eat it or not. You just feel a bit out of things… Leni

#### Theme 4 Limited comprehension

Theme 4, limited comprehension, explores the participants’ confusion and doubt about dietary self-management, especially in the early days following GDM diagnosis. Two sub-themes were identified: limited understanding of GDM, and limited understanding of GDM self-management requirements.

##### Limited understanding of GDM

In this sub-theme, participants expressed their confusion and lack of knowledge of GDM. This included a limited understanding of the importance of blood glucose control and dietary self-management. Here, Prani expresses her uncertainty, almost ten weeks after GDM diagnosis:

They didn’t tell me what’s the side effects for the baby. They just told me, “You’ve got diabetes, you’d better control with this and that.” But they didn’t tell me, like, what are the side effects for having sugar levels up… like why it’s really important to monitor your sugar levels… Prani

##### Limited understanding of GDM self-management requirements

Participants struggled to make sense of what they needed to do in order to maintain their blood glucose within the recommended range. This confusion was exacerbated by a limited amount of consistent information to guide their self-management efforts. Women described getting general dietary guidelines but insufficient information on 'how to' effect the necessary changes. This made the task more difficult and time consuming. Lili explains:

You get a list of food but no instructions… you don’t know “if I eat a smaller potato is that okay?” And then you follow it (the diet) through the pregnancy. …You would like to say, “Follow this diet, you’ll be fine, off you go.” It isn’t like that, so a greater effort is needed, and encouragement, and just well, time… you really have to work it out yourself… Lili

This difficulty was compounded by insufficient information about different foods and ingredients, and this was particularly the case for women who didn’t follow a Western diet. Tran explains:

I did get appointments where I got some information. But I found it more outside of those appointments. I mean it was helpful, but it wasn’t really in depth. Well like the food for instance, it wasn’t a very extensive list of what you could eat, it was very limited and most of the food I eat wasn’t on it… Tran

The information needs of women, in this study, varied and although many women felt the information they received was insufficient to help them manage their GDM successfully, others were happy with the depth of information they received while a small number of women felt that they received too much information:

Well the information they gave me was very basic so I looked up a lot on the internet and worked it out myself really…Tran

They give me a chart, like you can use these things [food] and you have to avoid these things. Exercise. They told me to do it at least 15 min exercise at the morning and evening. When I went to them they just give me information about how to control the diet. They told me everything… Gurtha

No at hospital they give me too much information about diabetes (indicates feeling overwhelmed)… Pina

#### Theme 5 Insulin as an easier option

Theme 5 explores the women’s commonly expressed belief that the use of insulin was an easier option, rather than dietary and exercise self-regulation alone. Women who struggled to adhere to the dietary regime were happy to commence on insulin as they felt it made their task more achievable. None of the women on insulin expressed any concern about long-term implications of insulin use and were mostly happy to accept it as a solution to the current situation.

Yeah, it’s painful, I have to say, but it really works. I have to say that is a good thing. Because it’s easier to help me manage my diet and control my sugar level… Yeah, the drugs (insulin) can help my condition …it was so hard to deal with cravings… Xioquan

#### Facilitators for GDM self-management

**Figure 2:**
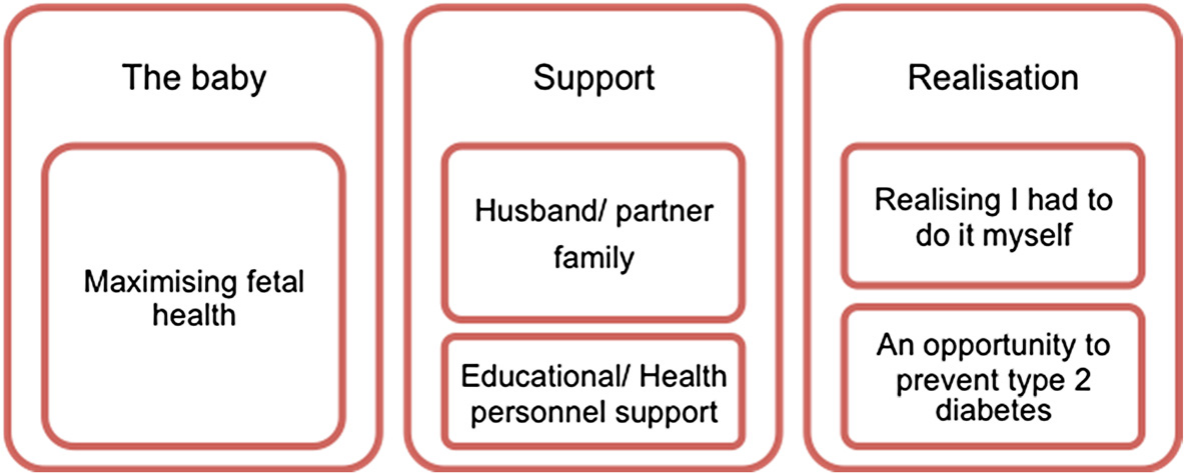
Facilitators of GDM self-management.

Factors identified as motivating and encouraging adherence to GDM self-management regimes included thinking about the baby, support from a variety of sources and realising that GDM self-management was in the woman’s hands. These three themes; (A) the baby, (B) support, and (C) realisation, are illustrated in Figure 2 below.

#### Theme A the baby

Women in this study had a powerful interest in maximising fetal health and this desire motivated them to avoid prohibited food items and to adhere to the GDM self-management regime, as closely as possible. Participants described being willing to do whatever they were required to do, in the baby’s best interests. Most understood clearly that GDM could impact negatively on the baby’s health. Xioquan explains:

It will affect your baby, so you have to do the right thing by the baby. I do have the temptation. But those times I control and I eat well for my baby because I be there for the baby… I do just monitor what I eat and, you know, more exercise definitely than before… Xioquan

Although participants were uniformly concerned about the baby’s welfare, and equally prepared to act in the baby’s best interests, their understanding of how diet and exercise would improve the baby’s health was sometimes a little sketchy:

Just cooking healthy food… and just walking, yeah, that makes the baby stronger…Fatima

Some women were additionally motivated by a strong desire to right earlier behaviours such as overeating and eating unhealthy foods. These women aimed to redress the balance in favour of the baby. Lili explains:

I was very determined to make sure I could do absolutely anything within my power to not allow any, something to happen to the baby. I knew I had brought it on myself by being overweight…I felt very responsible…Lili

#### Theme B support

Participants unanimously identified psychological support as very important in helping and encouraging them to master the everyday tasks of GDM self-management. This support had the effect of making the woman feel less isolated in her undertaking. Margaret explains:

Well I think I could, I probably could do it on my own but having that support base, having someone kind of do it with you … makes you feel you’re not alone doing it…Margaret

Support came from two key sources, the woman’s family and from health professionals such as dieticians, midwives, doctors and diabetic educators. Husbands and partners provided the most support and supported the women in terms of accompanying them on walks for exercise and encouraging them to adhere to the GDM diet as well as providing encouragement and emotional support when the woman was learning to take blood glucose levels and administer insulin. Several husbands/partners ate the same food, thus avoiding the need for the woman to cook separate meals. Some examples follow:

My husband, he was telling that I maybe … shouldn’t have that much [to eat], during the yum cha. … Xioquan

I was a bit fed up and upset with myself. My husband was very supportive… he helped me get over things like the finger pricks… he did his own one day just to show it wasn’t a big deal… Lili

He (husband) he’s fond of sweet. – now he eats, but not as much as before. He always – like, he try to control, as well… he won’t eat it in front of me… ‘cause whenever he, like, have sweet or other things, I want to eat…Gurtha

The woman’s mother or sisters were also a valuable source of support and encouragement, and mostly provided help in terms of advice about appropriate foods and ways of cooking food to reduce calorie content. Prani explains:

Yeah, especially my mother… she’s very worried about me, so she usually call(s) every day (from India). To find out, like, I’m okay. Don’t eat this. Don’t eat this… and she always try to give me some home recipes, so that I get through my diabetes… Prani

The second source of support was from health professionals, and this support was rated by the women as valuable, but as less important than family support. Educational support that improved the women’s comprehension and sense-making endeavours were valued most. Lili and Xioquan explain:

The first pregnancy I had a lady (educator) that was really excellent, very understanding, very approachable. She would listen to my concerns and we had a conversation rather than just a one-way flow of information. And so I had a very positive experience with her… Lili

The diabetes educator is really friendly…she explained things very like, in a very good way Yes, yes and – like, she did a demo in front of me, how to inject yourself. It was really scary first time… Xioquan

#### Theme C realisation

Women in this study described a stage of realising that they were responsible for their own care and that, other than the support that they harnessed from a variety of sources, essentially they had to do the work of GDM self-management alone. Two sub-themes were identified: realising I had to do it myself, and an opportunity to prevent type 2 diabetes.

##### Realising I had to do it myself

This sub-theme encompasses the women’s realisation that, although they could draw on family and health professionals for emotional and practical support, and also request assistance to develop management strategies, essentially the task of self-management fell to the individual woman. Leni and Lili explain:

‘Yeah, well you just have to do it yourself…you have to take it on… if you have it (GDM) you have it, you can’t do anything else’… Leni

I realised it was up to me…no one else… there was no point in cheating… I would be just cheating myself… Lili

##### An opportunity to prevent type 2 diabetes

The second sub-theme explores the women’s realisation that their GDM diagnosis offered them an opportunity to put in place strategies to prevent future type 2 diabetes. This realisation motivated the women to adhere to GDM management guidelines. Leanne’s account is typical:

It’s good to learn about it, otherwise the way I was going, definitely I would have diabetes 2. I didn’t know about it but now I can control myself and …Yeah, because the way I’m not having anything, I was having heaps of sugars every day …Leanne

## Discussion

This study aimed to explore the factors that facilitated or hindered GDM self-management among a group of women attending for pregnancy care in a low socio-economic setting. Findings suggest that women encountered a number of barriers in their quest to self-manage their condition. This included difficulty comprehending the urgency of immediate diet control. Most women spoke of the challenge of implementing a complex regimen of blood testing and dietary manipulation, within a very short time frame, while they were still coming to terms with the shock of diagnosis. Many reported commencing on insulin within 1–2 weeks of GDM diagnosis, and some women felt they would have mastered the requisite GDM self-management behaviours in a more generous time frame. This urgency of immediate treatment of maternal hyperglycaemia is echoed in the literature, where an immediate reduction of maternal blood glucose is recommended in order to minimize adverse pregnancy outcomes [13,49]. Moreover, recent studies also indicate that maternal hyperglycaemia, at lower levels that those previously recognised, has a detrimental effect on fetal welfare [25] and this finding has further increased pressure on health professionals to effect an immediate reduction in maternal blood glucose levels [13].

Participants in this study, found dietary self-management difficult, related to the time required to learn food values, and to cook healthy food. Social factors such as eating with family and friends also contributed to the dilemmas women faced, while a lack of clear guidelines was identified as hindering the process of diet control. Only two study participants succeeded in self-managing their GDM without insulin and both women, identified personal character strengths and determination as assisting them to master the necessary skills and behaviours. This very low rate of non-insulin use was a surprising finding, particularly as women were recruited on a first come basis rather than on the basis of management regimens. However, further explication of this finding is beyond the scope of this qualitative study of women’s experience and future quantitative evaluation is recommended. The finding may be incidental, however, it is consistent with generally higher use of insulin at the clinic where limited maternal education and understanding are thought to impact on poorer dietary adherence and higher rates of hyperglycaemia [19,20]. Whatever the reasons, rates of dietary self-management alone were considerably lower, among study participants than the recommended 65–90% of women discussed in the literature [24-26]. This feature may also reflect limited appropriate, culturally based educational resources for women in this area.

In general, dietary self-management is recognised as challenging [50,51] and as requiring motivation, understanding of food values and of the amount to eat [22]. This knowledge and motivation may have been deficient in our population due to their social circumstances and may have also been affected by cultural beliefs about particular foods, such as rice. Many participants struggled to believe that traditional foods such as rice could be considered ‘bad’ food, in terms of excess calories, related to portion sizes. Parallel findings present in the literature and dietary change is recognized as difficult to achieve, particularly among low socio-economic and migrant groups [52,53]. Such difficulties relate to cultural mores, views about traditional foods and a lack of appropriate food alternatives [50,51,53,54]. Many participants in our study were hesitant to change their diet, while at the same time they were willing to eat less in order to avoid hyperglycaemia. Parallel findings present in the literature, and participants in Rhoads-Baeza and Reis’ study among low income Latino women with GDM, were also reluctant to change from their traditional consumption of fatty meats to healthier alternatives [53]. On the other hand, Bandyopadhyay et al. [54] who studied South Asian women with GDM in Australia, found that participants predominantly changed to the recommended diet, but were nonetheless unhappy about the type and quantity of food allowed, and complained of always feeling hungry.

One surprising factor in this study, was the frequency with which women identified the use of insulin as an easier option, rather than dietary control alone. This finding is not evident in the literature and appears to relate to the women’s concerns about hyperglycaemia at the same time as encountering difficulties with dietary restrictions and behavioural change. Women who regarded insulin as easier than diet control alone, expressed limited concerns about insulin use and regarded it simply as a solution to their current dilemma of high blood glucose and difficulty in effecting diet control. None of these women displayed any knowledge of a possible link between insulin use in GDM and subsequent development of type 2 diabetes.

In terms of facilitators, women in this study were intensely interested in maximizing fetal health and this finding of concern for the fetus is echoed in other research on women’s experiences of GDM [53-55]. Concern for the fetus motivated participants to take on the tasks of GDM self-management and, although many women struggled to understand food values and to prepare healthy meals, they remained dedicated to the baby’s welfare. This manifested in the discomfort they endured by eating less than they desired, eating foods they did not enjoy, doing blood glucose levels and administering insulin, and trying to meet with exercise requirements. In the literature, a desire to protect the fetus, or evidence of maternal-fetal attachment, is similarly associated with greater pregnancy investment and adoption of health promoting behaviours, such as healthy diet [56,57].

Successful GDM self-management in our study was mediated by support from family and health professionals. Women identified husbands and partners as the most important source of psychological support. A less important, but additional form of psychological support was offered by health professionals, including diabetes educators, midwives, doctors, and dieticians. Similar findings of psychological support as important in encouraging GDM self-management, are found in the literature [58,59]. In particular, the partner’s support is seen as especially valuable in effecting behavioural change such as increasing exercise [59] while support from health professionals was recognised as encouraging women to view GDM as within their control [58].

Finally, this study has some limitations and the recruitment of women who could speak conversational English may have excluded many other migrant women in the area. For this reason, a number of interpreter mediated focus group discussions are planned for the future, which will include representation of the most populous ethnic groups in the area. Additionally, this small sample is from one geographical area, which means that the findings cannot be generalised to the Australian population as a whole [60]. However, the intent of the study was not to provide generalisable information, but to explore the facilitators or impediments to GDM self-management, among women in our area. This aim has been achieved and, although findings are not generalisable, they may also be applicable to other similar populations [60].

### Implications for practice

This study has important implications for practice, as rates of GDM continue to increase globally, particularly among women with risk factors such as obesity, lower socio-economic status and migration from world regions of high GDM risk. It is therefore important that strategies are adopted to encourage these groups of ‘at risk’ women to self-manage their GDM. Such self-management will reduce the incidence and severity of GDM related complications. The greatest challenge faced by health professionals, engaged in the care of women with GDM, is to provide sufficient and appropriate education and support at what is a stressful time in a woman’s pregnancy. Most women describe being shocked and upset at their diagnosis of GDM and take some time to adapt. At the same time, there is a relatively narrow window of opportunity for women to master the complex tasks of GDM self-management, and thus reduce their hyperglycaemia. Dwindling health resources add to this conundrum, as educational resources are already stretched, often where they are most needed.

There is a need for targeted educational resources for women with GDM, and earlier studies indicate that initiatives that address the cultural context of the group in question, may produce the best results [55,61]. Additionally, there is strong evidence to suggest that emotional support from the woman’s partner/husband/family improves adherence to GDM self-management regimens and, with this in mind, a family approach to GDM education may produce better results. This careful targeted approach may effect more successful dietary management and may thus reduce the percentage of women requiring insulin to control their condition. Successful GDM self-management, in turn, is associated with lower rates of serious pregnancy complication and serious infant morbidity. It is also associated with a lower risk of later developing type 2 diabetes.

## Conclusion

In conclusion, this study has indicated that women from low socio-economic and migrant backgrounds often struggle to comprehend and adhere to GDM dietary and exercise guidelines. They require supportive services that are culturally appropriate and pitched at an appropriate level of health literacy. A keen interest in the baby’s welfare is likely to increase women’s receptiveness to interventions.

## Competing interests

The authors declare that there are no competing interests.

## Authors’ contributions

Study conception and design, MC, GG, CS. Coordination and implementation of the study MC. Data collection MC. Data analysis, MC, GG, CS. Preparation of the manuscript MC. Editorial assistance, GG. All authors read and approved the final manuscript.

## Pre-publication history

The pre-publication history for this paper can be accessed here:

http://www.biomedcentral.com/1471-2393/12/99/prepub
